# Prognostic analysis of 2–5 cm diameter gastric stromal tumors with exogenous or endogenous growth

**DOI:** 10.1186/s12957-023-03006-9

**Published:** 2023-04-29

**Authors:** Chen Lin, Chao Sui, Tingting Tao, Wenxian Guan, Haoran Zhang, Liang Tao, Meng Wang, Feng Wang

**Affiliations:** 1 Department of General Surgery, Nanjing Drum Tower Hospital Clinical College of Nanjing University of Chinese Medicine, Nanjing, China; 2 Department of General Surgery, Nanjing Drum Tower Hospital, Affiliated Hospital of Medical School, Nanjing University, Nanjing, China

**Keywords:** Tumor growth pattern, Gastric stromal tumor, Progression-free survival, Overall survival

## Abstract

**Background:**

There has been limited research on the prognosis differences in patients with gastric stromal tumor invasion of the plasma membrane surface. This study intended to investigate whether there is a difference in prognosis in patients with endogenous or exogenous 2–5 cm diameter GISTs.

**Methods:**

We retrospectively analyzed the clinicopathological and follow-up data of gastric stromal tumor patients, all of whom underwent surgical resection for primary GIST at Nanjing Drum Tower Hospital from December 2010 to February 2022. We classified patients based on tumor growth patterns and then investigated the association between tumor growth patterns and clinical outcomes. Progression-free survival (PFS) and overall survival (OS) were calculated by the Kaplan‒Meier method.

**Results:**

A total of 496 gastric stromal tumor patients were enrolled in this study, among which 276 patients had tumors of 2–5 cm in diameter. Of these 276 patients, 193 had exogenous tumors, and 83 had endogenous tumors. Tumor growth patterns were significantly related to age, rupture status, resection style, tumor site, tumor size, and intraoperative bleeding. According to Kaplan‒Meier curve analysis, the tumor growth pattern among patients with 2–5 cm diameter tumors was significantly correlated with worse progression-free survival (PFS). Ultimately, multivariate analyses identified the Ki-67 index (*P* = 0.008), surgical history (*P* = 0.031), and resection style (*P* = 0.045) as independent prognostic markers for PFS.

**Conclusions:**

Although gastric stromal tumors with a diameter of 2–5 cm are classified as low risk, the prognosis is lower for exogenous tumors than for endogenous tumors, and exogenous gastric stromal tumors have a risk of recurrence. Consequently, clinicians should be vigilant regarding the prognosis of patients with this type of tumor.

## Introduction

Gastrointestinal stromal tumors (GISTs) are the most prevalent mesenchymal tumors in the gastrointestinal tract, with a primary occurrence in the stomach and secondary occurrence in the small intestine [1]. The annual incidence of GISTs is approximately 10–20 per million [2], and the median patient age associated with this incidence is approximately 60 years, with an equal distribution between sexes [3]. Pathogenetically, most gastrointestinal stromal tumors result from mutations in the KIT gene or platelet-derived growth factor receptor-*α* gene [4]. Patients with GISTs are increasingly being identified and treated in clinical practice, and surgical resection is the primary treatment, as GISTs are not responsive to radiotherapy or chemotherapy [5]. Tumor recurrence or progression in patients undergoing surgical resection is a significant challenge, with studies showing recurrence rates in approximately 50% of patients [6]. According to several analyses, tumor location, tumor size, and mitotic rate are important factors in assessing the prognostic outcome [7].

Gastric stromal tumors originate from the muscular layer of the gastric wall. According to their growth pattern, they are classified into exogenous or endogenous types. According to recent studies, the survival rate of patients with gastric stromal tumors is influenced by the location and size of the primary tumor [8]. According to the 2008 modified NIH risk classification, tumors are classified as knockdown risk, low risk, intermediate risk, and high risk [6]. Gastric stromal tumors with a diameter of 2–5 cm are classified as low risk. The current retrospective study found fewer cases of the endogenous type, which may be attributed to the safety of endoscopic resection for gastric stromal tumors smaller than 5 cm [9]. Most tumors less than 5 cm in diameter are of the endogenous type and are resected endoscopically. Numerous studies have investigated the correlations between biological behaviors and prognoses of gastric stromal tumors, including factors such as p53 [10], major cell types [11], tumor size [12], KIT mutation [13], cell density [14], and mitotic count [15]. Different tumor growth patterns have been studied in gastric stromal tumors and have been found to impact prognosis [16]. Our preliminary clinical study revealed that the level of serum CA125 can be used as an independent prognostic factor for gastrointestinal stromal tumors, providing new insights for clinical research [17]. This article discusses gastrointestinal stromal tumors (GISTs) with a diameter less than 5 cm, which predominantly exhibit an intraluminal growth pattern. In contrast, GISTs with a diameter between 5 and 10 cm exhibit both intraluminal and extraluminal growth patterns and can invade other organs. Up to 79% of GISTs demonstrate exogenous growth, while endogenous or mixed growth patterns are less common [18]. Our retrospective study analyzed patients with gastric stromal tumors with a diameter of 2–5 cm who were treated at Nanjing Drum Tower Hospital over a period of 12 years. We compared the clinicopathological characteristics and prognosis of endogenous and exogenous types of gastric stromal tumors to provide more guidance for clinicians in their treatment decisions.

## Methods and materials

### Patient section

**Fig. 1:**
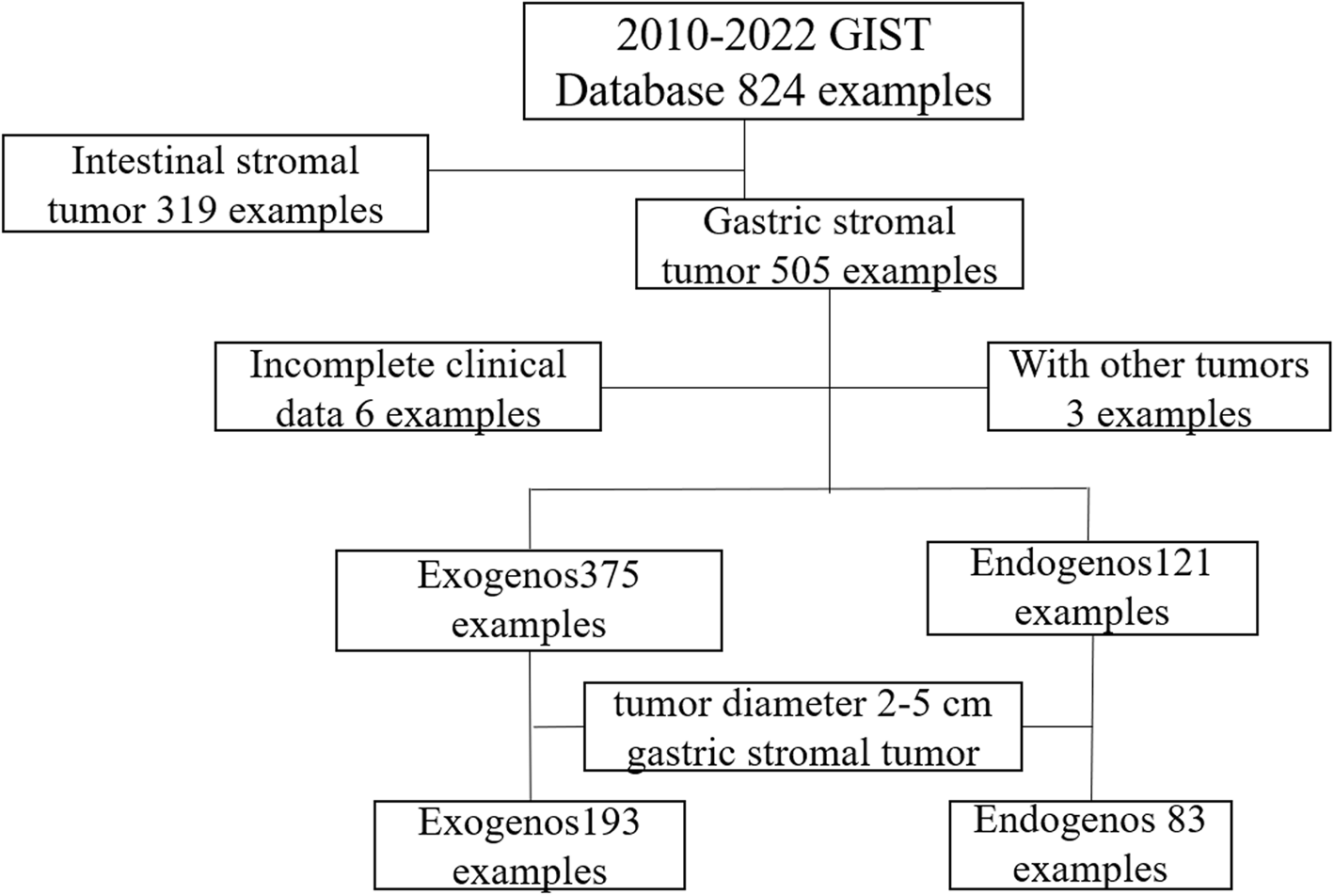
Flow chart of final patient admission

This study retrospectively analyzed the clinicopathological and follow-up data of GIST patients who underwent surgical resection at the Department of Gastrointestinal Surgery, Nanjing Drum Tower Hospital, from December 2010 to February 2022. The diagnosis of GIST relied on the Chinese and NCCN guidelines. The inclusion criteria were as follows: (1) 18–80 years old, (2) surgical resection, (3) GIST pathological diagnosis, (4) detailed and complete medical data, (5) no preoperative chemotherapy, and (6) no preoperative recurrence of metastases. The exclusion criteria were as follows: (1) patients with other concomitant carcinomas, (2) no surgical resection, (3) missing follow-up data, (4) patients who underwent emergency surgery, and (5) joint organ removal. Finally, a total of 493 patients were enrolled. The screening process is shown in Fig. 1.

### Study design

This was a single-center retrospective study. The primary outcome was PFS, which was defined as the time from the date of first surgery to the date of gastric stromal tumor progression or death. Overall survival (OS) was defined as the time from the first surgery date to the date of death. In addition, the date of the last follow-up visit was the study endpoint for PFS in the absence of progression or death. An endogenous growth pattern was defined as an intact intraoperative gastric plasma surface without abnormalities, while an exogenous growth pattern was defined as the observation of new growth during an intraoperative exploration of the gastric plasma membrane surface. Presurgical clinical data for patients at our hospital were collected by clinical physicians. Follow-up after surgery was conducted by specialized researchers using outpatient records and telephone interviews. Our follow-up visits included patients’ adjuvant treatment, recurrent metastases, and survival and postoperative reviews. In this study, we first grouped patients according to their tumor growth pattern and explored the relationship of this pattern with clinical prognosis. A total of 276 patients with gastric stromal tumors ranging in diameter from 2 to 5 cm were included, 193 of these patients had exogenous tumors, and 83 patients had endogenous tumors. We focused on whether the differences between endogenous and exogenous tumors were associated with gastric stromal tumor prognosis. Additionally, we examined laboratory tests, pathological data, and other factors to identify their impact on the prognosis of patients with gastric stromal tumors.

### Statistical analysis

SPSS 25.0 software (IBM Corporation, Armonk, NY, USA). The measurements were compared by independent samples *t*-test, while categorical variables were compared using the *χ*^2^ test or Fisher’s exact test. Examples (%) were used to express statistical information. Kaplan‒Meier analysis was used to plot the survival curves of the two groups, and the log-rank method was used to analyze the difference in survival rates between the two groups. Univariate and multivariate Cox proportional-hazard regression model analyses were used to identify independent factors for the recurrence of gastric stromal tumors. A *P*-value of less than 0.05 was considered to indicate statistical significance.

## Results

### Patient characteristics

Among the 496 patients initially included in this study, 375 had exogenous tumors, and 121 had endogenous tumors. The clinicopathological parameters between the two groups are compared in Table 1, which shows a significant difference in rupture status (*P* = 0.001), age (*P* = 0.023), resection style (*P* = 0.026), tumor site (*P* = 0.013), tumor size (*P* = 0.002), and intraoperative bleeding (*P* = 0.030). These findings indicate that the tumor growth pattern is associated with the tumor size. Gastric stromal tumors are categorized by size into three groups: < 2 cm, 2–5 cm, and > 5 cm. Tumors with a diameter of < 2 cm are considered to have a very low risk of recurrence and patient death, while those with a diameter of > 5 cm are considered to have a moderate to high risk and receive more attention from clinicians. Tumors with a diameter of 2–5 cm are traditionally classified as low risk, but they still pose a risk of recurrence, and clinicians may not pay enough attention to them [18]. Therefore, we selected gastric stromal tumors of 2–5 cm diameter for this retrospective study. Among the 276 patients with tumors of this size, 193 had exogenous tumors, and 83 had endogenous tumors. The clinicopathological parameters between the two groups are compared in Table 2, which shows a significant age difference (*P* = 0.002) and HB (*P* = 0.005). However, the growth pattern of tumors 2–5 cm in diameter was not associated with clinical characteristics, including mitotic index, imatinib treatment, patient sex, tumor site, and NIH risk grade.

**Table 1:** Association between tumor growth patterns and clinicopathological features

Characteristics	Exogenous (*n* = 375)	Endogenous (*n* = 121)	*p*-value
**Age (%)**			**0.023**
≤ 65	270 (76.1)	75 (61.9)	
> 65	105 (23.9)	46 (38.1)	
**Gender (%)**			0.987
Female	210 (43.5)	67 (55.4)	
Male	165 (56.5)	54 (44.6)	
**Rupture (%)**			**0.001**
No	370 (94.1)	120 (99.2)	
Yes	5 (5.9)	1 (0.8)	
**Tumor site (%)**			**0.013**
Small bend	130 (34.7)	38 (31.4)	
Large bend	156 (41.6)	60 (49.6)	
Fundus	89 (23.7)	23 (19.0)	
**Tumor size (%)**			**0.002**
< 5 cm	37 (9.8)	11 (9.1)	
2–5 cm	192 (51.2)	83 (68.6)	
> 5 cm	146 (39.0)	27 (22.3)	
**Mitotic index (%)**			0.403
≤ 5/HPF	268 (71.5)	92 (76.0)	
> 5/HPF	107 (28.5)	29 (24.0)	
**NIH risk grade (%)**			0.057
Extremely low or low	189 (50.4)	76 (62.8)	
Moderate or high	186 (49.6)	45 (37.2)	
**CD117 (%)**			0.775
( −) or ( +)	103 (27.5)	29 (24.0)	
(+ +) or (+ + +)	272 (72.5)	95 (76.0)	
**CD34 (%)**			0.696
( −) or ( +)	69 (18.4)	12 (10.0)	
(+ +) or (+ + +)	306 (81.6)	109 (90.0)	
**Surgical history (%)**	308 (82.1)	95 (78.5)	0.451
**HB (X̄)**	127	124	0.124
**ALB (X̄)**	40.4	39.95	0.172
**Adjuvant imatinib (%)**	279 (74.4)	87 (72.0)	0.671
**Basic disease (%)**	215 (57.3)	68 (56.2)	0.909
**Resection style (%)**			**0.026**
Mesenchymal resection	331 (88.3)	96 (79.3)	
Distal gastrectomy	14 (3.7)	12 (10.0)	
Proximal gastrectomy	18 (4.8)	10 (8.3)	
Total gastrectomy	12 (3.2)	3 (2.4)	
**Surgery time (min)**	120	115	0.884
**Intraoperative bleeding(ml)**	50	50	**0.030**

**Table 2:** Association between 2 and 5 cm tumor growth patterns and clinicopathological features

Characteristics	Exogenous (*n* = 193)	Endogenous (*n* = 83)	*p*-value
**Age (%)**			**0.002**
≤ 65	147 (76.1)	49 (75.9)	
> 65	46 (23.9)	34 (24.1)	
**Gender (%)**			0.412
Female	84 (43.5)	52 (62.6)	
Male	109(56.5)	31 (37.4)	
**GI bleeding (%)**			0.082
No	143 (78.8)	61 (73.5)	
Yes	50 (21.2)	18 (26.5)	
**Tumor site (%)**			0.052
Small bend	72 (37.3)	44 (53.0)	
Large bend	73 (37.8)	23 (27.8)	
Fundus	48 (24.9)	16 (19.2)	
**Adjuvant imatinib (%)**	121 (46.2)	62 (23.7)	0.307
**Mitotic index (%)**			0.660
≤ 5/HPF	192 (70.1)	51 (63.0)	
> 5/HPF	82 (29.9)	30 (37.0)	
**NIH risk grade (%)**			0.248
Extremely low or low	157 (81.3)	64 (77.1)	
Moderate or high	36 (18.7)	19 (22.9)	
**CD117 (%)**			0.071
( −) or ( +)	55 (28.5)	18 (21.7)	
(+ +) or (+ + +)	138 (71.7)	65 (78.3)	
**CD34 (%)**			0.286
( −) or ( +)	48 (24.9)	10 (12.1)	
(+ +) or (+ + +)	138 (71.5)	73(87.9)	
**Ki-67 index (%)**			0.372
< 5%	155 (80.3)	64 (77.1)	
≥ 5%	38 (19.7)	19 (22.9)	
**HB (X̄)**	130	121	**0.005**
**ALB (X̄)**	40.3	39.7	0.078
**Resection style (%)**			0.200
Mesenchymal resection	179 (64.9%)	70 (25.4%)	
Distal gastrectomy	6 (2.2%)	7 (2.5%)	
Proximal gastrectomy	6 (2.2%)	5 (1.8%)	
Total gastrectomy	1 (0.4%)	1 (0.4%)	
**Basic disease (%)**	87 (31.5%)	43 (15.6%)	0.370
**Surgical history (%)**	37 (13.4%)	19 (6.9%)	0.588

### Impact of exogenous and endogenous factors on prognosis

We regarded the tumor growth pattern as a variable in the Cox proportional-hazard regression model. Univariate analysis showed that resection style (*P* = 0.041, *HR* = 0.121, 95% *CI*: (0.016 ~ 0.914); tumor growth pattern (*P* = 0.048, *HR* = 0.130, 95% *CI*: 0.017 ~ 0.986); Ki-67 index (*P* = 0.005, *HR* = 1.206, 95% *CI*: 1.057 ~ 1.376); and surgical history (*P* = 0.049, *HR* = 2.914, 95% *CI*: 1.004 ~ 8.461) were significantly associated with PFS (Table 3). Subsequently, multivariate analysis demonstrated that resection style (*P* = 0.045, *HR* = 2.015, 95% *CI*: 0.190 ~ 14.438), Ki-67 index (*P* = 0.008, *HR* = 1.070, 95% *CI*: 1.018 ~ 1.125), and surgical history (*P* = 0.031, *HR* = 3.066, 95% *CI*: 1.110 ~ 8.474) were independent predictive factors for PFS (Table 3).

**Table 3:** Cox proportional-hazard regression model analysis for PFS

Factors	Univariate analysis PFS		Multivariate analysis PFS	
	HR (95% *CI*)	*p*-value	HR (95% *CI*)	*p*-value
**Age**		0.070		
≤ 65	Reference			
> 65	1.041 (0.997 ~ 1.086)			
**Gender**		0.659		
Male	Reference			
Female	1.234 (0.385 ~ 4.412)			
**GI bleeding**				
No	Reference	0.520		
Yes	1.374 (0.521 ~ 3.625)			
**Growth pattern**		**0.048**		0.058
Exogenous	Reference		Reference	
Endogenous	0.130 (0.017 ~ 0.986)		7.070 (0.933 ~ 53.595)	
**Tumor site**		0.887		
Small bend				
Large bend	Reference			
Fundus	1.046 (0.562 ~ 1.945)			
**Resection style**		**0.041**		**0.045**
Mesenchymal resection	Reference		Reference	
Distal gastrectomy	0.121 (0.016 ~ 0.914)		2.015 (0.190 ~ 14.438)	
Proximal gastrectomy				
Total gastrectomy				
**Mitotic index**		0.802		
≤ 5/HPF	Reference			
> 5/HPF	1.261 (0.206 ~ 7.699)			
**NIH risk grade**		0.798		
Extremely low or low	Reference			
Moderate or high	0.841 (0.224 ~ 3.162)			
**CD117**		0.747		
( −) or ( +)	Reference			
(+ +) or (+ + +)	0.590 (0.024 ~ 14.513)			
**CD34**		0.813		
( −) or ( +)	Reference			
(+ +) or (+ + +)	1.471 (0.060 ~ 36.235)			
**Ki-67 index**		**0.005**		**0.008**
≤ 5%	Reference		Reference	
> 5%	1.206 (1.057 ~ 1.376)		1.070 (1.018 ~ 1.125)	
**Basic disease**	Reference	0.788		
	1.144 (0.429 ~ 3.049)			
**Surgical history**	Reference	**0.049**	Reference	**0.031**
	2.914 (1.004 ~ 8.461)		3.066 (1.110 ~ 8.474)	
**Adjuvant imatinib**	Reference	0.278		
	2.035 (0.564 ~ 7.342)			

Table 4 shows that GI bleeding (*P* = 0.044, *HR* = 0.293, 95% *CI*: 0.088 ~ 0.968) and basic disease (*P* = 0.036, *HR* = 0.276, 95% *CI*: 0.083 ~ 0.921) were significantly correlated with OS by univariate analysis. The multivariate analysis showed no independent risk factors for OS (Table 4).

**Table 4:** Cox proportional-hazard regression model analysis for OS

Factors	Univariate analysis RFS		Multivariate analysis RFS	
	HR (95% *CI*)	*p*-value	HR (95% *CI*)	*p*-value
**Age**		0.162		
≤ 65	Reference			
> 65	0.951 (0.886 ~ 1.020)			
**Gender**		0.614		
Male	Reference			
Female	0.672 (0.144 ~ 3.144)			
**GI bleeding**				
No	Reference	**0.044**	Reference	0.179
Yes	0.293 (0.088 ~ 0.968)		− 1.348 (− 14.456 ~ 2.707)	
**Growth pattern**		0.938		
Exogenous	Reference			
Endogenous	0.000 (0.000 ~ 7.291E)			
**Tumor site**		0.839		
Small bend				
Large bend	Reference			
Fundus	1.099 (0.442 ~ 2.734)			
**Resection style**		0.695		
Mesenchymal resection	Reference			
Distal gastrectomy	0.728 (0.149 ~ 3.757)			
Proximal gastrectomy				
Total gastrectomy				
**Mitotic index**		0.128		
≤ 5/HPF	Reference			
> 5/HPF	0.001 (0.000 ~ 8.248)			
**NIH risk grade**		0.117		
Extremely low or low	Reference			
Moderate or high	50.034 (0.224 ~ 3.162)			
**CD117**		0.925		
( −) or ( +)	Reference			
(+ +) or (+ + +)	0.849 (0.027 ~ 26.498)			
**CD34**		0.249		
( −) or ( +)	Reference			
(+ +) or (+ + +)	7.801 (0.237 ~ 257.082)			
**Ki-67 index**		0.806		
≤ 5%	Reference			
> 5%	0.970 (0.760 ~ 1.237)			
**Basic disease**	Reference	**0.036**	Reference	0.588
	0.276 (0.083 ~ 0.921)		0.558 (− 7.005 ~ 12.538)	
**Surgical history**	Reference	0.693		
	1.363 (0.293 ~ 6.348)			
**Adjuvant imatinib**	Reference	0.686		
	1.289 (0.378 ~ 4.397)			

Overall, the tumor growth pattern was found to be an independent risk factor for PFS (Table 3) but not for OS (Table 4).

### Prognosis comparison between the two groups

**Fig. 2:**
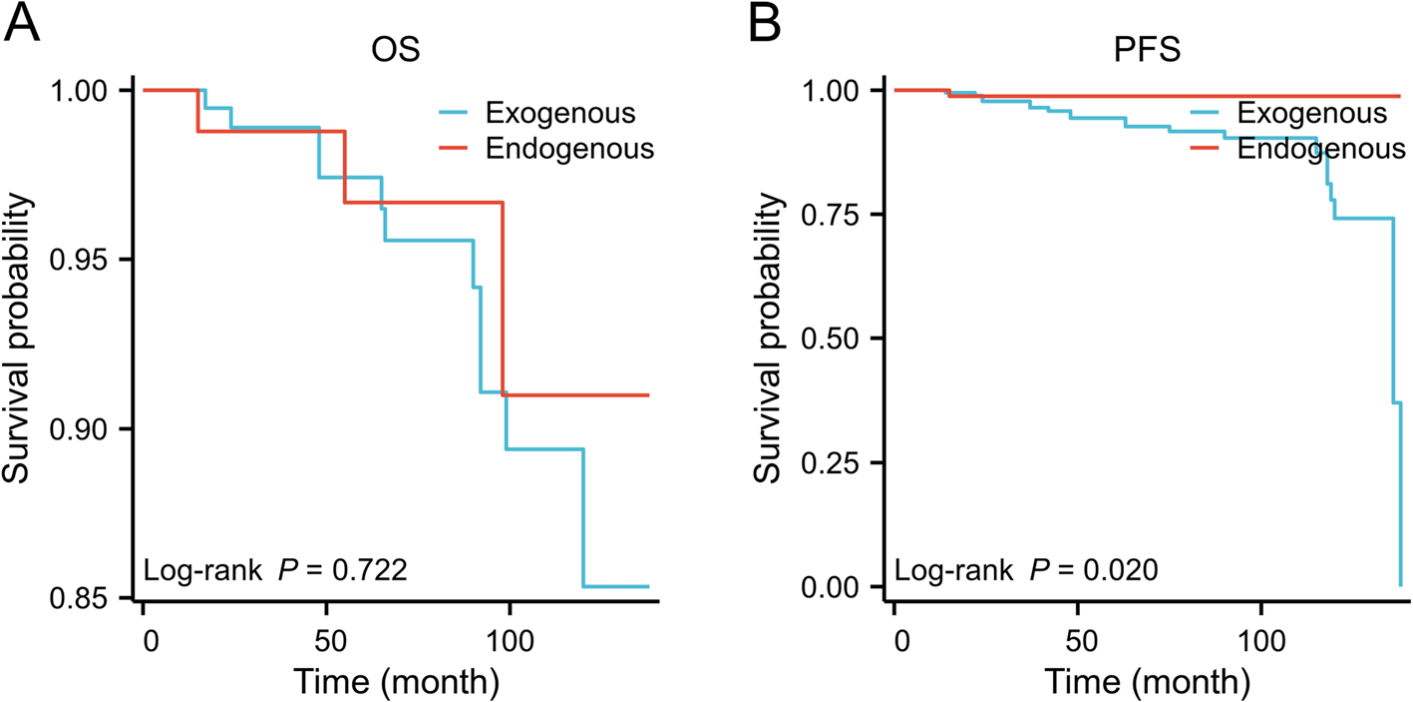
Kaplan–Meier curves of PFS and OS with the exogenous and endogenous gastric stromal tumors 2–5 cm in diameter. **A** OS. **B** PFS

Follow-up of these 276 patients ranged from 12 to 148 months. At the time of the last follow-up (Feb 2022), 21 patients were found to have gastric stromal tumors, and 14 patients were dead. The median follow-up time for this study was 33 months, with a median follow-up time of 80 months. According to Kaplan‒Meier curve analysis, tumor growth patterns (*P* = 0.021) were associated with PFS in patients with gastric stromal tumors (Fig. 2B). The PFS for patients with exogenous 2–5 cm gastric stromal tumors was 89.6%. The PFS for patients with endogenous 2–5 cm gastric stromal tumors was 98.7%. A *P*-value of less than 0.05 was considered to indicate statistical significance.

In a subsequent study of the effect of tumor growth patterns on OS (*P* = 0.722), we found no association between the two in the 276 patients with gastric stromal tumors we studied (Fig. 2A). Fewer patients with 2–5 cm diameter gastric stromal tumors died. The OS of patients with exogenous 2–5 cm gastric stromal tumors was 99%. The OS of patients with endogenous 2–5 cm gastric stromal tumors was 99%. A *P*-value above 0.05 was considered to indicate no statistical significance.

## Discussion

This study was a single-center retrospective analysis. We retrospectively analyzed the clinicopathological data of 276 patients with 2–5 cm diameter gastric stromal tumors treated at Nanjing Drum Tower Hospital. Gastrointestinal stromal tumors are common mesenchymal tumors of the gastrointestinal tract. In recent years, with the development of diagnostic techniques, the diagnosis rate of gastric stromal tumors has gradually increased [19].

This study intended to explore the influencing factors of the clinical outcomes in Chinese patients with 2–5 cm diameter gastric stromal tumors with different tumor growth patterns through the integration and analysis of clinical pathological data. We focused on 2–5 cm diameter gastric stromal tumors to elucidate the exact relationship between tumor growth patterns and patient prognosis by combining preoperative laboratory tests and postoperative reexamination data. To the best of our knowledge, this research established a connection between tumor growth patterns and 2–5 cm diameter gastric stromal tumors for the first time.

In this study, we used PFS and OS as outcome measures and found that tumor growth patterns had a close relationship with worse PFS through Kaplan‒Meier curve analysis. Subsequently, we demonstrated that tumor growth patterns were independent risk factors for PFS, but similar results were not observed in the multivariate analysis of OS. In univariate analysis, bleeding and underlying diseases were considered prognostic factors for overall survival. However, in a more precise multivariate analysis, no independent prognostic factors related to overall survival were found. Additionally, there was no correlation between overall survival and the growth pattern (*P* = 0.722) of gastric stromal tumors (Table 4). A recent study examined the association between surface ulceration in gastrointestinal stromal tumors and their growth patterns, revealing a strong correlation between the two [20]. Our study showed the significance of resection style, Ki-67 index, and surgical history on PFS in patients with endogenous and exogenous tumors. Gastric stromal tumors tend to grow swollen in the submucosa and rarely involve lymph nodes [21]. Most gastric stromal tumors are endogenous, so most gastric mesenchymal tumors are resected surgically using endoscopy for relatively large-diameter tumors. Research also shows that full laparotomy is used when the endogenous type of tumor is in the lesser curvature in the stomach. The location of the endophytic tumor is unclear using plasmacytoma [22]. According to the 2019 NCCN guidelines, experienced surgeons are advised to opt for laparoscopic surgery in favorable sites such as the greater curvature of the stomach [23]. In this study, there were 96 cases of tumors in the large bend, 116 in the small bend, and 64 in the fundus (Table 2). In this retrospective study, we found that differences in surgical approach were factors affecting PFS in patients with direct 2–5 cm gastric stromal tumors. Ki67, also known as MKI67, is a proliferation-associated nuclear marker of tumor cells [24]. Gumurdulu et al. found that Ki67 was useful as a prognostic factor along with tumor size, mitotic index, and tumor grade [25]. In a previous study, Ki-67 was shown to be significantly associated with prognosis and tumor recurrence in GISTs [26]. Surgical resection is the primary treatment for localized disease and is the preferred approach for GISTs [27]. Ki67 in this study was likewise an independent factor that influenced PFS in patients with direct 2–5 cm gastric stromal tumors. Our study found that surgical history was also an important influencing factor for PFS, and patients with a history of surgery had a worse prognosis than those without a history of surgery. This study included 56 patients with surgical histories (Table 2).

The study had several limitations. First, this research was a retrospective examination, which means that selection bias cannot be completely avoided. Second, it only included a single-center cohort, and further external validation is required to demonstrate whether the present results are feasible for other patient cohorts. Thus, multicenter prospective studies are necessary to further clarify the relationship between different tumor growth patterns and prognosis in patients with 2–5 cm diameter gastric stromal tumors. Despite these limitations, the study provides valuable insights into clinical practice and highlights the need for clinicians to pay more attention to exogenous 2–5 cm diameter gastric mesenchymal tumors due to their higher risk of recurrence.

## Conclusion

In conclusion, this retrospective study provides important insights into the relationship between tumor growth patterns and clinical outcomes in patients with 2–5 cm diameter gastric stromal tumors. Our results suggest that tumor growth patterns are independent predictors of progression-free survival, and that the exogenous type is associated with a significantly worse prognosis than the endogenous type. Therefore, we conclude that exogenous 2–5 cm diameter gastric stromal tumors have a high risk of recurrence and require the attention of clinicians.

## Data Availability

The data are not publicly available due to privacy or ethical restrictions. Access to the data and the calculation method can be obtained from the corresponding author by email fengwang36@163.com.
